# Bridging sponsor–site operations through reciprocal on-site training of CRAs and CRCs: Impact on workflow knowledge and behavior among trial staff

**DOI:** 10.1017/cts.2026.10694

**Published:** 2026-02-04

**Authors:** Hiroyuki Hosono, Nao Moriyama, Tatsuki Mukoyama, Yoshiaki Kariya, Yuriko Nishimura, Kensuke Yamada, Yuri Ishida, Rumi Kudo, Kazumi Yabe, Masashi Uchida, Takeo Fukagawa

**Affiliations:** 1 Education Center for Medical Pharmaceutics, Graduate School of Pharmaceutical Sciences, The University of Tokyo, Japan; 2 Center for Clinical Research & Trial, Teikyo University Hospital, Japan; 3 Clinical Trial Monitoring Department, Bristol-Myers Squibb K.K., Japan; 4 Laboratory of Pharmaceutical Regulatory Sciences, Graduate School of Pharmaceutical Sciences, The University of Tokyo, Japan; 5 Department of Pharmacy, The University of Tokyo Hospital, Faculty of Medicine, The University of Tokyo, Japan; 6 Department of Surgery, Teikyo University School of Medicine, Japan

**Keywords:** Clinical trials, clinical research associate (CRA), clinical research coordinator (CRC), reciprocal on-site training, team science

## Abstract

**Background:**

Opportunities for face-to-face interaction between sponsor-side clinical research associates (CRAs) and site-side clinical research coordinators (CRCs) have decreased with remote and risk-based monitoring, potentially impeding communication and mutual understanding – key determinants of team functioning. Accordingly, we implemented a reciprocal on-site training to enhance CRA-CRC mutual understanding and evaluated its impact.

**Methods:**

Seventeen sponsor staff, including 11 CRAs, joined an 8-hour hospital tour with CRC-guided process simulations and discussion; conversely, 14 hospital staff, including 11 CRCs, attended a 4-hour sponsor-office visit with system demonstrations and discussion. Self-assessed understanding of counterpart workflows and impressions of the counterpart group were rated pre- and post-training on 5-point Likert scales. Free-text feedback underwent text-mining analysis. Behavioral change was surveyed 6 months later.

**Results:**

CRAs improved on all 9 understanding items (e.g. “flow of daily medical practice:” median score 2.0 vs. 4.0, pre- and post-training, respectively, *p* < 0.0001); CRCs improved on 4 of 5. Positive impressions increased and negative impressions decreased in both groups (e.g. “bright atmosphere:” median 3.0 vs. 5.0 for CRAs, *p* = 0.0002; 3.0 vs. 5.0 for CRCs, *p* = 0.0044). Text-mining revealed the specific content participants learned, which included keywords reflecting this training’s objective of enhancing mutual understanding. At 6 months, 70% of CRAs and 88% of CRCs reported changes in their work behavior.

**Conclusions:**

A brief, reciprocal, on-site training improved CRA–CRC mutual understanding and perceptions, with sustained self-reported behavioral changes in work practices. From a team science perspective, such practical training may strengthen sponsor-site communication and collaboration.

## Introduction

In the pharmaceutical development process, the compliant and efficient conduct of clinical trials is essential for generating high-quality evidence. Various clinical research professionals (CRPs), including principal investigators (PIs), play important roles in ensuring trial success. As most sponsored clinical trials are conducted at medical institutions, clinical research associates (CRAs), representing the sponsor, and clinical research coordinators (CRCs), representing the trial sites, serve as the primary communication interface between the sponsor and the trial site. Together, they collaborate as parts of a team to implement specific trial protocols [1].

CRAs, also known as clinical trial monitors, are typically affiliated with the sponsor or a Contract Research Organization (CRO) acting on the sponsor’s behalf. Their responsibilities include site feasibility assessment, site initiation, staff training, on-site monitoring, and ensuring data quality and regulatory compliance [2]. In contrast, CRCs – also referred to as clinical research nurses (CRNs), clinical trial nurses (CTNs), research nurse coordinators (RNCs), or study coordinators – are site-based personnel involved in trial operations. In Japan, the term “CRC” is commonly used to describe them as they are from diverse healthcare backgrounds such as nursing, pharmacy, and clinical laboratory science [3]. CRCs are employed either by the investigative site or by Site Management Organizations (SMOs). They are responsible for coordinating trial preparations, liaising with related departments and sponsors, supporting informed consent, managing participant care and addressing data queries from sponsors, and play a role in supporting the PIs and sub-investigators to ensure the smooth progress of clinical trials [4].

CRAs and CRCs are pivotal to the execution of clinical trials. It is emphasized that clear role delineation, ongoing training, and collaborative approaches are essential for the successful conduct of clinical trials and the advancement of medical research [1,5]. Since clinical trials require multidisciplinary operational teams composed of diverse CRPs including CRAs and CRCs, the application of a team science perspective is critical to considerations of clinical trial conduct [6]. The team science literature identifies team-based communication as a core determinant of performance in translational science teams [7].

Despite sharing the common goal of conducting compliant trials, CRAs and CRCs operate within distinct organizational cultures – CRAs within the corporate, metrics-driven environment of sponsors, and CRCs within the participant-care-focused setting of healthcare institutions. This gap can lead to misaligned expectations and communication challenges. A Tufts Center for the Study of Drug Development (CSDD) survey highlighted that investigative sites often experience poor communication flow and a lack of respect from sponsor side [8]. Given that the frequency of CRA site visits has decreased due to the adoption of remote monitoring and risk-based monitoring (RBM) and replacement of on-site pre-selection and site initiation visits with remote modalities [9,10], the resulting reduction in direct CRA–CRC contact may impede communication and reduce opportunities to organically learn about each other’s work situations and priorities beyond what is typically captured during formal monitoring or feasibility visits. Accordingly, the establishment of effective communication and collaboration at the operational level has become a recognized challenge.

To address this challenge, experiential training programs that bring together CRAs and CRCs to learn about each other’s operational workflows and role responsibilities within their organizational context may be an effective approach. However, reports evaluating the impact of such training are limited both in Japan and internationally, as most existing studies have focused on site-level GCP training, protocol-specific education or stay-interviews between staff and leadership [11], with little attention to interprofessional experiential programs targeting mutual understanding among CRPs [6]. Therefore, we designed and implemented an on-site, experiential training program with the goal of fostering mutual understanding between CRAs and CRCs. This study examines the program’s effectiveness by assessing key outcomes: (1) change in self-assessed understanding of workflows, roles and organizational structures, (2) shift in mutual perceptions, (3) qualitative insights gained, and (4) self-reported changes in work behavior. This paper aims to propose a novel strategy for improving CRA–CRC collaboration through mutual understanding and perceptions.

## Methods

### Implementation of the training program

We conducted a reciprocal, on-site experiential training program in which clinical trial professionals affiliated with trial sponsors (hereafter referred to as “CRA group”) and those affiliated with trial sites (hereafter referred to as “CRC group”) visited each other’s respective workplaces. All participants were staff members engaged in sponsor–site communication and collaboration. The CRA group visited a university hospital functioning as a clinical trial site to observe the daily operations of the CRC group, while the CRC group visited the offices of a pharmaceutical company acting as the trial sponsor to observe the CRA group’s operations. During the site visits, one or two experienced staff members from the host institution, who also are the investigators of this study, accompanied the participants, providing real-time explanations and responding to questions regarding their respective roles. Following the tours, reflection and exchange sessions were held between CRA and CRC participants to facilitate discussion and exchange of perspectives. The training program was implemented under a confidentiality agreement signed by Teikyo University Hospital, Bristol-Myers Squibb K.K. and The University of Tokyo.

Trainers from both the sponsor organization and the university hospital comprised of (i) investigators of this study who served in that role on a self-nominated basis and (ii) staff currently working in the respective roles who were invited by the investigators. Likewise, CRAs and CRCs enrolled as participants through self-nomination rather than managerial assignment. Although some CRAs and CRCs worked on the same clinical study, formal pairing was not required; participants also included individuals engaged in different projects. All training was conducted between November 2023 and July 2024.

#### CRA training (hospital visit program)

The CRA group received training at Teikyo University Hospital that had entered into a contract with their company (Bristol-Myers Squibb K.K.) to conduct ongoing clinical trials. The training program lasted approximately 8 hours and followed a standardized agenda (Table 1), and the program was held three times, one for each participant cohort, with the same content. The CRA training was led by the head CRC of the university hospital’s CRC team, who also provided the opening remarks. During the ”Hospital Tour and Discussion” session, staff members from each department involved in trial operations (e.g., nurses, pharmacists, clinical laboratory technologists), as well as experienced CRCs (with more than 10 years of experience), explained both routine and trial-specific tasks in detail. CRA participants also followed the movement pathways of clinical trial participants and CRCs within the hospital. In “CRC role explanation and simulation” and “Investigational drug management” (in the “Introduction to Center for Clinical Trials” session), participants engaged with a trial-specific process sheet used at Teikyo University Hospital. Using an initial investigational drug administration visit as an example, CRCs demonstrated the sequence of preparation activities and the physical movement of trial participants and CRCs within the hospital. These process sheets are created for each trial to clarify workflows, source documentation practices, and quality control procedures. Participants also attended the “Clinical trial admin and IRB functions” session that addressed site administrative operations at the sponsor–site interface, including contract negotiation and execution, regulatory oversight, and institutional review board (IRB) procedures (e.g., submissions, approvals, continuing review, and reporting pathways). The program did not include any interaction with clinical trial participants. In the “Reflection and Exchange” session, CRA and CRC participants – matched in number – gathered in a meeting room and engaged in open discussion. Discussion topics emerged spontaneously from the reflections on the training experience.

**Table 1.** Agenda for CRA training (hospital visit program)

Time	Training content
∼30 min	**Welcome and overview** - Opening remarks - Explanation of training objectives
∼2 hours	**Introduction to Center for Clinical Trials** - Organizational structure - Clinical trial admin and IRB functions - CRC role explanation and simulation - Investigational drug management - Q&A
∼4 hours	**Hospital Tour and Discussion** **Dermatology outpatient clinic:** - Patient flow and consultation environment - Observation of consultation rooms and back areas during clinic hours **Inpatient ward (general ward supporting cardiovascular ICU):** - Observation of patient movement and environment - Tour of staff station, professional roles, infection control, etc. **Pharmacy department:** - Workflow of investigational drug dispensing (oral, topical, injectable) - Staff roles and communication pathways **Central laboratory:** - Blood/urine sampling procedures for trial participants - Observation of laboratory devices - Overview of in-house and outsourced testing workflow **Center for Clinical Trials:** - Tour of drug storage and document archive - Explanation of task allocation, document/information handling
∼1.5 hours	**Reflection and Exchange** - Small group and open discussion - Feedback and wrap-up

CRA = clinical research associate; CRC = clinical research coordinator; ICU = intensive care unit; IRB = institutional review board.

#### CRC training (sponsor visit program)

The CRC group’s training was conducted at the sponsor (Bristol-Myers Squibb K.K.)’s corporate office for CRCs affiliated with Teikyo University Hospital, conducting the sponsor’s trials. The training program followed a predefined agenda (Table 2), lasted approximately 4 hours, and was conducted twice, one for each participant cohort, with the same content. The CRC training was led by the manager of the sponsor’s monitoring department, who also provided the opening remarks. During the “Office Tour and Q&A” session, sponsor staff guided participants through workspaces and meeting areas, addressing questions throughout. The “Introduction to Sponsor Operations and Q&A” session included presentations by CRAs and other clinical operations personnels on the roles and interdepartmental collaborations within the sponsor organization. Live Q&A was encouraged throughout. In the “Reflection and Exchange” session, CRC and CRA participants – approximately equal in number – gathered in a meeting space to engage in open discussion, with themes emerging organically from training reflections.

**Table 2.** Agenda for CRC training (sponsor visit program)

Time	Training content
∼15 min	**Welcome and overview** - Opening remarks - Explanation of training objectives
∼1 hour	**Office Tour and Q&A** **Areas visited:** - Workspace (free-desking) - Meeting rooms - Remote SDV room - Cafeteria - Document archive - Document dispatch/receiving area
∼3 hours	**Introduction to Sponsor Operations and Q&A** **Topics covered:** - Organizational structure and clinical trial implementation framework: In-house study process Site selection process Outsourced study process CRO oversight - Systems and document management: CTMS (Clinical Trial Management System) eTMF (electronic Trial Master File) EDC (Electronic Data Capture) Contract management system Tracking sheets (site procedures, essential documents) Handover forms for site personnel Time and goal management system Expense reimbursement system e-Learning system Metrics dashboard - Education for operations staff/monitors - Protocol development and vendor selection - Data management and query handling - Informed consent form management - Adverse event reporting: SAE (Serious Adverse Event) process Aggregate safety reporting
∼30 min	**Reflection and Exchange** - Group discussion and feedback session

CRC = clinical research coordinator; CRO = Contract Research Organization; IRB = institutional review board; SDV = source document verification.

For the CRA training, the hospital tour component could not be conducted with a large cohort at one time; therefore, the CRA program was delivered in 3 smaller cohorts, while CRC program was delivered in 2 cohorts. Session length and core content were held constant across cohorts.

### Evaluation approach

To assess the effectiveness of the training program in achieving its goal of enhancing mutual understanding, we measured the following outcomes: (1) change in self-assessed understanding of counterpart’s workflows, roles, and organizational structures, (2) shift in mutual perceptions, (3) qualitative insights gained from the training (via open-ended responses), and (4) self-reported behavioral changes in work practices. To evaluate the outcomes of (1) and (2), we administered pre- and post-training surveys to both CRA and CRC participants. The post-training surveys were conducted within one week of each participant’s training. In the post-training survey, open-ended questionnaires were also conducted for investigation of the outcome of (3). The surveys were conducted anonymously using Google Forms, configured to avoid collecting email addresses. To enable comparison between pre- and post-training responses without personal identification, participants were asked to enter the same self-generated pseudonym (nickname) in both surveys. The survey settings did not allow participants to view their pre-training responses at the time of completing the post-training survey.

Approximately 6 months after the overall training series (i.e., 6 months after the last training, not 6 months after each individual’s training), we conducted a follow-up survey to assess the outcome of (4). This anonymous Google Form survey did not request pseudonyms and thus could not be linked to prior responses.

The survey instruments were jointly developed by representatives of both the clinical trial site and the sponsor, all of whom served as members of the investigator team. Participation in the surveys was voluntary. Participants were informed that their responses would remain confidential and would not influence any work-related evaluations.

#### Pre- and post-training surveys

**Figure 1.**
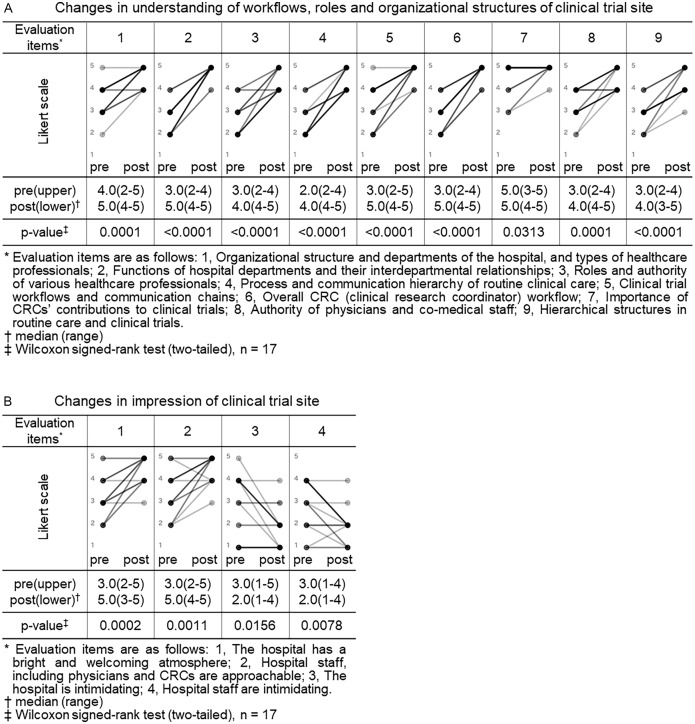
Changes in understanding of workflows, roles and organizational structures and impressions of clinical trial site (CRA (clinical research associate) group). Pre- and post-training self-assessment scores are presented by dots. Individual participant trajectories are represented by lines connecting pre- and post-training scores. The intensity of the grayscale (from gray to black) reflects the density of overlapping data points and lines, with darker shading representing a larger number of participants with identical scores or patterns of change.

**Figure 2.**
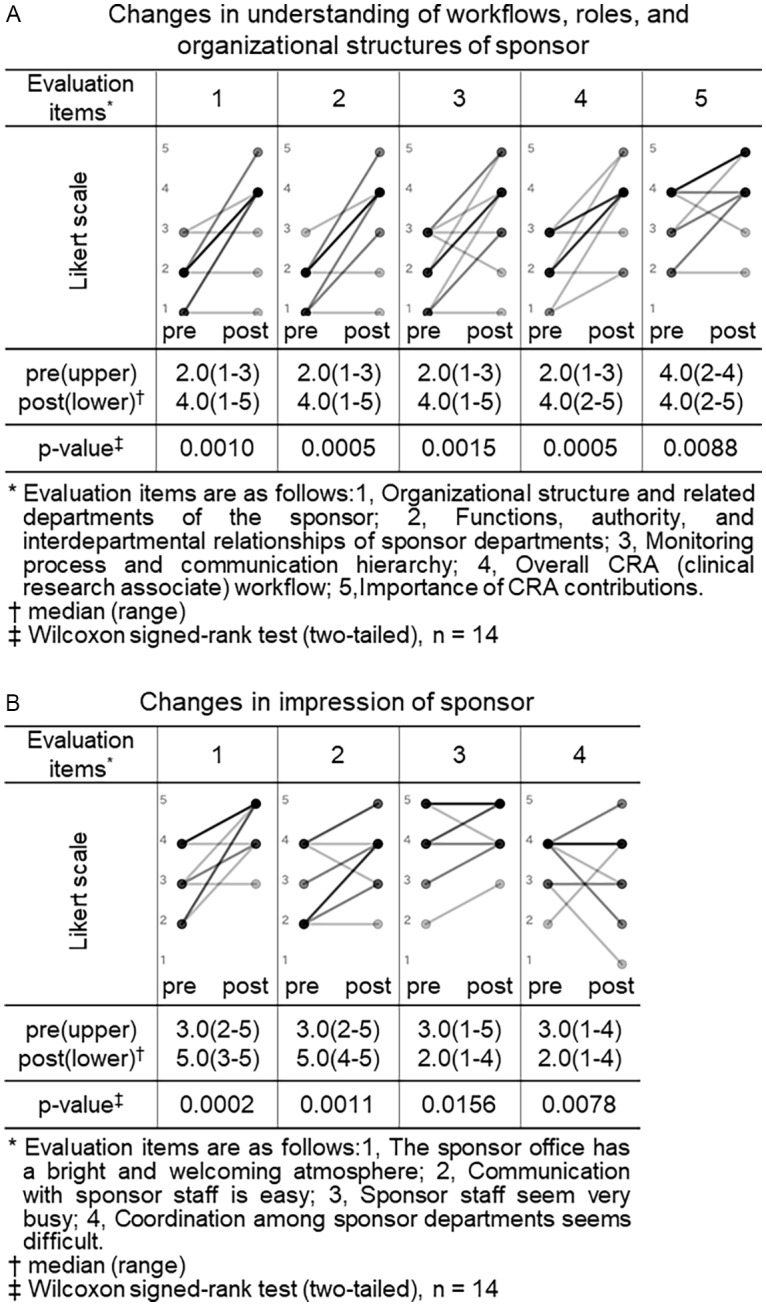
Changes in understanding of workflows, roles and organizational structures and impressions of sponsor (CRC (clinical research coordinator) group). Pre- and post-training self-assessment scores are presented by dots. Individual participant trajectories are represented by lines connecting pre- and post-training scores. The intensity of the grayscale (from gray to black) reflects the density of overlapping data points and lines, with darker shading representing a larger number of participants with identical scores or patterns of change.

Both the CRA and CRC participants completed pre- and post-training surveys. All closed-ended items used a 5-point Likert scale (1 = not at all/strongly disagree; 5 = very well/strongly agree). For knowledge/understanding, CRA participants self-rated their understanding regarding trial site across 9 items (as shown in legend of Figure 1, Panel A), and CRC participants self-rated their understanding regarding sponsor across 5 items (as shown in legend of Figure 2, Panel A). For perceptions/impressions, both groups rated four items pertaining to the counterpart organization (as shown in legends of Figures 1 and 2, Panels B).

Background characteristics were also collected. For CRAs, these included:(1) age group (20s–30s or 40s–60s), (2) experience visiting trial sites as a CRA, (3) prior work experience at medical institutions, (4) academic background (healthcare-related, other science-related, or other), and (5) experience with hospital training during university. For CRCs, (1) age group (20s–30s or 40s–60s), (2) experience visiting a sponsor company, (3) prior work experience at sponsor organization or CRO, and (4) professional background.

#### Post-training open-ended questionnaire

At the same time of post-training survey, both CRA and CRC participants answered the same five open-ended questions:

1. New insights gained through the training.
2. Elements considered useful for improving future practices.
3. Things learned about the staff at the host institution for the first time.
4. Aspects of the training that could influence your own practice.
5. General impressions or feedback about the training program.

#### Follow-up survey on behavioral changes in work practices

Approximately six months after the overall training series, all participants were invited to complete an anonymous Google Form assessing whether or not any behavioral changes in work practices had occurred during study start-up (pre-initiation) and during trial implementation (post-initiation). Participants who reported behavioral changes in work practices were asked to provide examples through open-ended responses.

### Data analysis process

#### Comparison of understanding and impressions pre- and post-training

Scores for self-assessed understanding of counterpart roles and overall impressions (rated on a 5-point Likert scale from 1 to 5) were compared between pre- and post-training responses for both the CRA and CRC groups. The Wilcoxon signed-rank test was used for paired comparisons between the two time points. A p-value of less than 0.05 was considered statistically significant. All statistical analyses were performed using JMP Pro 18 (SAS Institute Inc., Tokyo, Japan). In the graphical representations of these changes (Figures 1 and 2), individual pre- and post-training scores are connected by lines, with darker shading indicating a greater density of overlapping data points and lines.

#### Quantitative text analysis of open-ended responses post-training

To complement the Likert-scale–based self-evaluation, we conducted a quantitative text analysis of participants’ post-training open-ended responses. In quantitative text analysis conducted with text-mining software, the text was segmented into words, after which statistical procedures are applied to estimate term frequencies and term–term associations (co-occurrences), which are then displayed in visual summaries. Compared with a stand-alone thematic analysis, this method offers such advantages as (i) comprehensive coverage, because all comments are included in analysis, and (ii) objectivity and reproducibility, because terms and their relations are computed by standardized algorithms, thereby reducing analyst bias. We then interpreted the prominent terms and co-occurrence patterns in light of their original textual contexts, integrating qualitative meaning with quantitative patterns. We adopted this mixed (quantitative–qualitative) strategy to explore, with minimal preconceptions, what participants learned and to examine their insights regarding CRA–CRC mutual understanding and perceptions.

Specifically, we analyzed the CRA and CRC responses collectively, excluding the item of “General impressions or feedback about the training program,” which deviates from the purpose of this analysis. The analysis was performed, as previously described, using KH Coder 3.02c official package (SCREEN Advanced System Solutions Co., Ltd., Kyoto, Japan), a widely used text-mining software for quantitative content analysis, originally designed for analysis of Japanese-language text data [12–14]. Briefly, after text cleansing, we created a custom inclusion list of technical terms to improve extraction accuracy (e.g., “CRC,” “CRA,” “trial site”). In parallel, to ensure analytical focus, we manually inspected the top 100 most frequent terms. Through discussion, two investigators reached a consensus to exclude 16 terms thar were deemed context-specific, non-analytical words (e.g., “usual,” “learning,” “training”) as well as common Japanese words with low thematic specificity (e.g., “think,” “feel,” “know”) to prevent artificial connections between unrelated topics. Terms that captured specific nuances of participant insights (e.g., “abundant,” “wondering”) were retained for the final co-occurrence network analysis.

We applied the “co-occurrence network” module of KH Coder, as previously described [12–14], to identify themes in the free-text data – a common quantitative content analysis technique that detects thematic clusters from frequently co-occurring terms. The number of subgraphs (clusters) was determined automatically by the software’s modularity optimization algorithm, ensuring an empirically based structure independent of researcher subjectivity. To interpret each subgraph, we examined KWIC (Key Words in Context) concordances for the terms within that subgraph to review their usage in the original sentences. Using these contexts, we extracted representative quotations for each subgraph and derived its qualitative meaning, thereby characterizing the overall landscape of the open-ended responses.

Based on the representative quotations, we then qualitatively explored what participants learned and the insights they gained regarding CRA–CRC mutual understanding, perceptions, and communication. The interpretation of the generated subgraphs and the qualitative exploration of participants’ insights were conducted independently by two investigators of this study. They subsequently discussed their independent interpretations to ensure validity and finalized by consensus.

#### Analysis of behavioral change survey

For the follow-up survey conducted approximately six months after the overall training series, we summarized the presence or absence of reported behavioral changes in work practices separately for the CRA and CRC groups. Additionally, we reviewed participants’ open-ended responses to understand the nature of these behavioral changes in detail.

## Results

### Implementation of the training

For the CRA training (hospital visit program) at Teikyo University Hospital, a total of 17 staff members from Bristol-Myers Squibb K.K. participated. These 17 participants were divided into 3 distinct cohorts, which included 5, 6, and 6 individuals. The program outlined in Table 1 was delivered three times, one for each cohort, with the same content. The CRA group consisted of 11 CRAs, 2 clinical trial managers (CTMs), 1 global trial manager (GTM), 1 clinical trial associate (CTA), 1 regional clinical compliance lead (RCCL), and 1 study start-up specialist (SSUS). None of the participants had prior work experience in a medical institution. In terms of age distribution, 12 participants were in their 20s–30s and 5 were in their 40s–60s. Regarding academic background, 10 participants had graduated from healthcare-related faculties, 6 from other science-related faculties, and 1 from a non-science faculty. Among the healthcare-related graduates, 8 had completed some form of hospital-based practical training as students. All sponsor participants had current or prior experience working as CRAs.

For the CRC training (sponsor visit program) at the offices of Bristol-Myers Squibb K.K., a total of 14 staff members from Teikyo University Hospital participated. These 14 participants were divided into 2 distinct cohorts, consisting of 7 individuals each. The program outlined in Table 2 was delivered twice, one for each cohort, with the same content. The CRC group included 11 CRCs and 3 administrative staff from the trial coordination office. Age distribution included 8 participants in their 20s–30s and 6 in their 40s–60s. None had experience visiting a sponsor company, and none reported prior work experience at a sponsor organization or CRO. The professional backgrounds of participants were as follows: 8 pharmacists, 2 nurses, 1 clinical laboratory technologist, 1 physiotherapist, and 2 others. All site participants, with the exception of one administrator, had current or prior experience working as CRCs.

### Survey responses

The pre- and post-training surveys and open-ended questionnaires yielded a total of 17 (100%) responses from the CRA group and 14 (100%) from the CRC group, matching the number of participants. For the follow-up behavioral change survey conducted approximately 6 months after the overall training series, 12 (71%) responses were obtained from the CRA group and 9 (64%) from the CRC group.

### Comparison of pre- and post-training understanding and impressions

#### CRA group

We compared the CRA group’s pre- and post-training self-assessment scores to evaluate changes in their understanding of workflows, roles and organizational structures and their impressions of clinical trial site (Figure 1).

Eight of nine items related to understanding of clinical trial site showed a statistically significant increase in post-training self-assessment scores compared to pre-training (*p* < 0.05, Wilcoxon signed-rank test). Notable improvements were observed in items such as “functions of hospital departments and their interdepartmental relationships” (median score: pre 3.0 vs. post 5.0, *p* < 0.0001), “process and communication hierarchy of routine clinical care” (median: 2.0 vs. 4.0, *p* < 0.0001), and “overall CRC workflow” (median: 3.0 vs. 5.0, *p* < 0.0001). Even for the item “importance of CRCs’ contributions to clinical trials,” which had relatively high scores before the training, a statistically significant change was observed (median: 5.0 vs. 5.0, *p* = 0.0313), indicating increased consensus or confidence (Figure 1, panel A).

All 4 items related to impressions of clinical trial site also showed statistically significant changes. Positive impressions improved significantly, such as “the hospital has a bright and welcoming atmosphere” (median pre, 3.0 vs. post, 5.0, *p* = 0.0002) and “hospital staff including physicians and CRCs are approachable” (3.0 vs. 5.0, *p* = 0.0011). In contrast, negative impressions decreased significantly: “the hospital is intimidating” (3.0 vs. 2.0, *p* = 0.0156) and “hospital staff are intimidating” (3.0 vs. 2.0, *p* = 0.0078) (Figure 1, panel B). In Japan, hospitals are sometimes perceived as unfriendly or intimidating settings, characterized by quiet, impersonal environments and also as places people visit when anxious about illness. In addition, historically hierarchical clinician–patient dynamics may convey asymmetry. Therefore, we assessed not only staff demeanor but also the broader atmosphere of the hospital to capture contextual factors that might hinder sponsor–site communication and mutual understanding and to examine whether the program could shift these impressions.

#### CRC group

We similarly compared the CRC group’s pre- and post-training self-assessment scores to evaluate changes in their understanding of workflows, roles and organizational structures, and impressions of sponsor organizations (Figure 2).

Among the 5 items assessing understanding of sponsor, four showed statistically significant increases in median scores after training: “organizational structure and related departments of the sponsor” (2.0 vs. 4.0, *p* = 0.0010), “functions, authority, and interdepartmental relationships of sponsor departments” (2.0 vs. 4.0, *p* = 0.0005), “monitoring process and communication hierarchy” (2.0 vs. 4.0, *p* = 0.0015), and “overall CRA workflow” (2.0 vs. 4.0, *p* = 0.0005). Although the item “importance of CRA contributions” had a consistent median score of 4.0 before and after training, the change was statistically significant (*p* = 0.0088), suggesting a shift in distribution or increased agreement among respondents (Figure 2, panel A).

Among the 4 items assessing impressions of sponsor organizations, two showed significant improvements: “the sponsor has a bright and welcoming atmosphere” (3.0 vs. 5.0, *p* = 0.0005) and “communication with sponsor staff is easy” (2.5 vs. 4.0, *p* = 0.0044). In contrast, no significant changes were observed for the items “sponsor staff seem very busy” (4.0 vs. 5.0, *p* = 0.125) and “coordination among sponsor departments seems difficult” (4.0 vs. 4.0, *p* = 0.5625) (Figure 2, panel B).

### Quantitative text analysis of post-training open-ended responses

**Figure 3.**
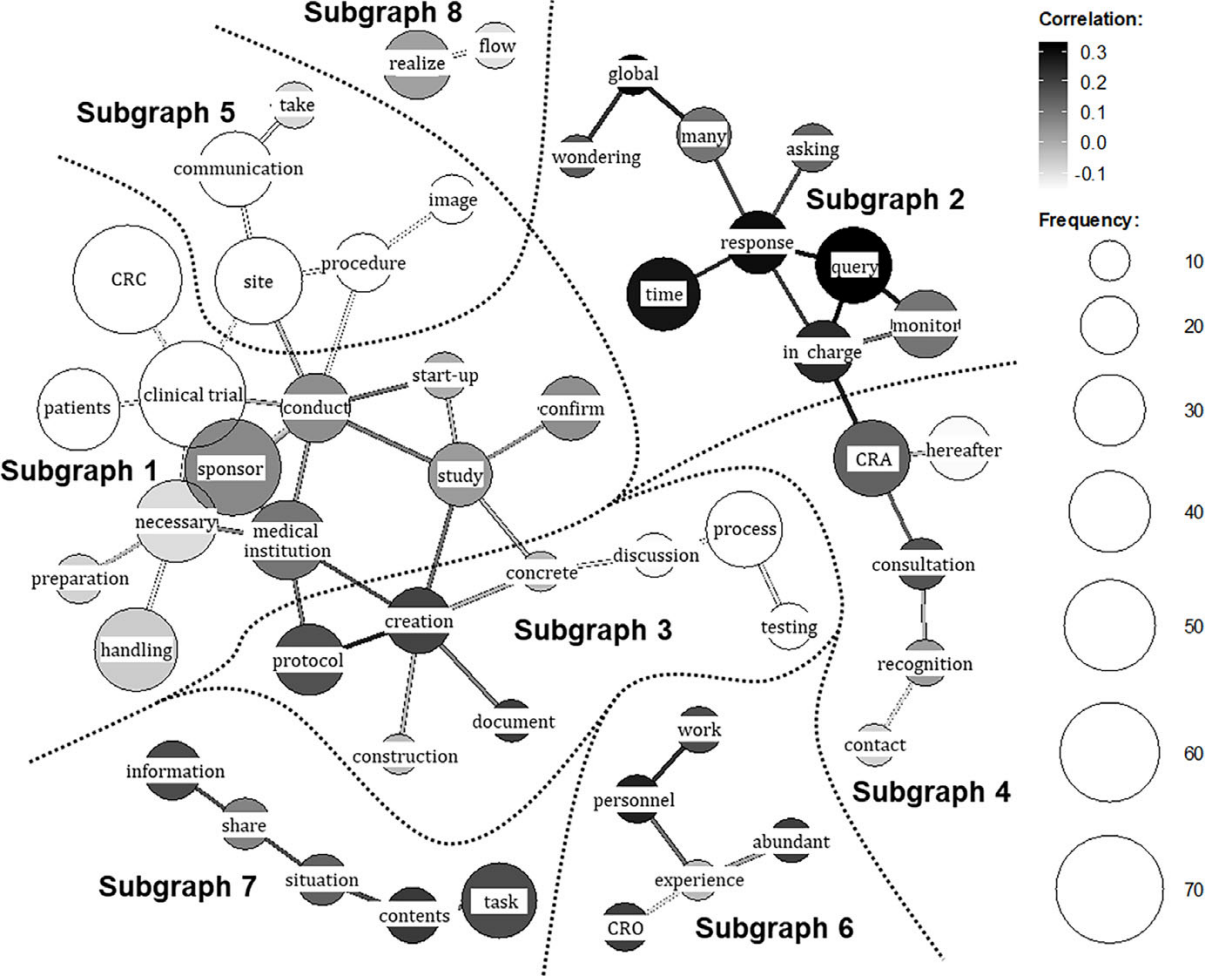
Co-occurrence network of key terms from post-training open-ended responses. The network visualizes frequently co-occurring keywords extracted from the qualitative responses of both CRA (clinical research associate) and CRC (clinical research coordinator) participants regarding insights gained. The respondent group (CRA or CRC) is indicated by grayscale shading, with lighter tones denoting nodes (frequently used words) and edges (relationships) more characteristic of CRA responses and darker tones those more characteristic of CRC responses. The subgraphs extracted by the analysis are enclosed with dashed lines for visual clarity. The themes derived for the subgraphs are as follows: ***Subgraph 1***, CRA and CRC recognition of the counterpart’s operational context and priorities; ***Subgraph 2***, CRC understanding of CRA operations, particularly query handling; ***Subgraph 3***, CRA and CRC understanding of protocol development and execution; ***Subgraph 4***, CRA and CRC understanding of contact points; ***Subgraph 5***, CRA reflections on communication with CRCs and evolving perceptions of CRCs; ***Subgraph 6***, CRC understanding of the CRO (Contract Research Organization) role; ***Subgraph 7***, CRA and CRC perspectives on intra-organizational information sharing; ***Subgraph 8***, CRA and CRC understanding of workflow.

Figure 3 presents the co-occurrence network generated through the text-mining technique. In this visualization, frequently used words (nodes) with strong co-occurrence relationships are connected by lines (edges), enabling an overview of the open-ended responses through inspection of connected word groups. Distinct subgraphs (clusters of strongly connected words) were identified based on the network structure. The respondent group (CRA or CRC) is indicated by grayscale shading, with lighter tones denoting words (nodes) and relationships (edges) more characteristic of CRA responses and darker tones those more characteristic of CRC responses. In total, eight subgraphs were extracted.

To interpret each subgraph, KWIC (Key Word in Context) concordances for the terms within the cluster were examined, and representative quotations were extracted and summarized in Table 3. By integrating these quotations with the network structure, the following qualitative meanings were derived for the subgraphs in Figure 3: Subgraph 1 – CRA and CRC recognition of the counterpart’s operational context and priorities (representative responses: Table 3: ⟨1⟩–⟨6⟩); Subgraph 2 – CRC understanding of CRA operations, particularly query handling (Table 3: ⟨7⟩–⟨8⟩); Subgraph 3 – CRA and CRC understanding of protocol development and execution (Table 3: ⟨9⟩–⟨10⟩); Subgraph 4 – CRA and CRC understanding of contact points (Table 3: ⟨11⟩–⟨12⟩); Subgraph 5 – CRA reflections on communication with CRCs and evolving perceptions of CRCs (Table 3: ⟨13⟩); Subgraph 6 – CRC understanding of the CRO role (Table 3: ⟨14⟩); Subgraph 7 – CRA and CRC perspectives on intra-organizational information sharing (Table 3: ⟨15⟩–⟨16⟩); and Subgraph 8 – CRA and CRC understanding of workflow (Table 3: ⟨17⟩–⟨18⟩). Thus, a comprehensive grasp of the entire landscape of the open-ended responses was obtained.

**Table 3.** Examples of open-ended responses containing key terms from each subgraph

Subgraph	Examples of open-ended responses*
Subgraph 1: *CRA and CRC recognition of the counterpart’s operational context and priorities*	<1> [CRA] I was able to understand what it really means for clinical trials to be part of routine care. I realized how difficult it is to complete participant visits without mistakes and without inconveniencing the participants, especially when every department is so busy. I want to communicate with the medical institution staff with this in mind. <2> [CRA] What left a strong impression was that CRCs are busier than I imagined – planning, preparing, moving around, and coordinating with others when participants come in. <3> [CRA] I came to recognize that both CRCs and CRAs want to communicate and cooperate in conducting clinical trials. <4> [CRC] I usually work in an environment where communication tends to happen one-on-one between CRA and CRC, but I was reminded that conducting clinical trials rely on the support of many other people. <5> [CRC] The work I, as a CRC, am handling right now is filled with the input and expectations of many people on the sponsor side. <6> [CRC] I learned that there are many timelines set by the sponsor, and that from the site side, it’s important to handle as early as possible for efficiency.
Subgraph 2: *CRC understanding of CRA operations, particularly query handling*	<7> [CRC] I’m glad I learned specific solutions, like noting the reason when a query cannot be resolved, or having the CRA consult directly with the CTM. <8> [CRC] I learned that status updates are shared frequently. However, the explanations also helped me realize again that it takes time to get responses due to global checks and meeting frequency.
Subgraph 3: *CRA and CRC understanding of protocol development and execution*	<9> [CRA] By actually seeing the process of participants moving from reception to consultation and lab testing, I learned how hard it is for participants to move around, and how important CRC support is. Understanding the parts of CRC work that are normally hidden will help me more clearly visualize and comprehend CRC activities more concretely during future discussions. <10> [CRC] I learned for the first time that there are many steps involved in protocol creation. Knowing that, I felt it’s natural for sponsors to want sites to start up quickly.
Subgraph 4: *CRA and CRC understanding of contact points*	<11> [CRA] I was able to learn about internal hospital movements and coordination with other departments, and how some of these steps are required by sponsor requests – activities I hadn’t been aware of as a CRA. I should contact and communicate with those considerations in mind hereafter. <12> [CRC] I recognized that even issues considered minor by the site may be frequent issues or critical to trial conduct, and that consulting with CRA about them not as complaints but for system-wide improvement could be meaningful.
Subgraph 5: C*RA reflections on communication with CRCs and evolving perceptions of CRCs*	<13> [CRA] I had unconsciously viewed site staff as intimidating and was hesitant to communicate with them, but the reality was more relaxed than I expected, so I feel like I can ask more freely from now on.
Subgraph 6: *CRC understanding of the CRO (Contract Research Organization) role*	<14> [CRC] I learned about the relationship and coordination between CRAs and CROs, and now I feel I can better judge which personnel to consult based on the issue, how long it may take to resolve, and so on.
Subgraph 7: *CRA and CRC perspectives on intra-organizational information sharing*	<15> [CRA] I’m now able to clearly visualize the workflows and coordination of each department, so when I make a request as a CRA, I’ll be sure to communicate in a way that reflects the recipient’s perspective and helps them visualize the task. <16> [CRC] I learned that regular internal team meetings are held and information is shared between sites, so I felt it’s important to give detailed progress reports and issues to the CRA.
Subgraph 8:*CRA and CRC understanding of workflow*	<17> [CRA] I learned about the flow of trial participants going through consultations and exams and understood that trials are conducted in hospitals that also handle many other patients. <18> [CRC] I realized that even though our roles are different, we’re part of one work flow of new drug development, so I felt it’s okay to speak openly with each other.

*Note:* *[CRA]: An example of CRA group responses; [CRC]: An example of CRC group responses.

CRA = clinical research associate; CRC = clinical research coordinator; CTM = clinical trial manager.

Qualitative exploration of the representative quotations was then conducted as outlined in the Methods, by synthesizing the interpretations of these eight subgraphs (specific topics), we qualitatively identified four broader, overarching insights regarding what participants learned: (i) comprehension of the counterpart’s workflows; (ii) recognition of role division and consultation pathways; (iii) appreciation of the counterpart’s professional role and priorities; and (iv) awareness of communication and information-sharing strategies.

Regarding (i), CRAs reported a clearer grasp of internal hospital flows including medical examinations, testing, specimen transport and site workload intensity (Table 3: ⟨2⟩, ⟨9⟩, ⟨17⟩). Conversely, CRCs gained understanding of sponsor-side structures, including global approval pathways and the involvement of multiple departments (Table 3: ⟨8⟩; ⟨4⟩, ⟨10⟩). Recognition of each other’s operational burdens appeared in both groups with a foundation for more considerate and effective communication (Table 3: ⟨11⟩, ⟨16⟩).

With respect to (ii), CRCs reported clearer consultation pathways (e.g., CRA or CTM) for issues such as queries (Table 3: ⟨7⟩, ⟨14⟩), whereas CRAs described greater attention to institutional procedures and workload when making requests (Table 3: ⟨15⟩).

For (iii) and (iv), CRAs recognized site-side optimization of workflows to ensure participant safety alongside clinical efficiency (Table 3: ⟨1⟩), while CRCs gained insight into sponsor responsibilities for regulatory compliance and quality across global timelines (Table 3: ⟨5⟩, ⟨6⟩). This shared understanding of each other’s priorities was associated with a shift from oppositional to collaborative stances in problem solving (Table 3: ⟨12⟩) and with reduced psychological barriers to communication (Table 3: ⟨3⟩, ⟨13⟩, ⟨18⟩), fostering expectations of deeper future interaction.

### Survey on behavioral change in work practices

Approximately six months after the overall training series, a follow-up survey was conducted to assess whether participants had experienced any behavioral changes in work practices as a result of the program. Responses were obtained from 71% of CRA group participants (12 out of 17) and 64% of CRC group participants (9 out of 14). For the study start-up phase (prior to trial initiation), 70% of CRA respondents (7 out of 10) and 88% of CRC respondents (7 out of 8) reported changes in their behavior in work practices. Participants who were not engaged in relevant tasks at the time of the survey were excluded from the denominator. During the trial conduct phase (after study initiation), 67% of CRA respondents (8 out of 12) and 100% of CRC respondents (9 out of 9) reported behavioral changes in work practices.

Examples of reported changes included the following:

- “When reviewing processes, I’ve started to visualize how participants and CRCs move through the site.” (CRA group)
- “I now more frequently confirm the differences between routine clinical workflows and trial workflows with the site, and I find it easier to understand the reasons behind site staff’s questions.” (CRA group)
- “When I encounter difficulty answering queries in the EDC, I used to ask the CRA right away, but now I try to think through the problem myself. As a result, the time it takes to resolve queries has decreased.” (CRC group)
- “In trials with poor enrollment, I now actively share with the sponsor not only whether there are eligible participants, but also why we were unable to proceed with explanation or informed consent.” (CRC group)

## Discussion

This study found that a reciprocal, on-site training program achieved its goal of enhancing mutual understanding between CRAs and CRCs. In the Likert-scale data, participants showed statistically significant improvements in self-assessed understanding of each other’s workflows, roles and organizational structures, accompanied by more favorable impressions. The qualitative analysis further indicated a clearer appreciation of counterparts’ processes and priorities, recognition of role division and consultation pathways, and heightened awareness of communication and information-sharing strategies. Approximately six months after the program, participants also reported positive behavioral changes in their work practices, suggesting a durable, team science-consistent impact on sponsor-site collaboration.

Team science is defined as “scientific collaboration, i.e., research conducted by more than one individual in an interdependent fashion, including research conducted by small teams and larger groups” by The National Research Council [15]. Historically, research on team science has predominantly focused on translational researchers – scientists integrating theories and methodologies from various disciplines [16]. This line of research has identified barriers to effective collaboration and proposed tools to overcome them. In team-based translational settings, for instance, Kelly *et al.* propose a “Translational Team Science Hierarchy of Needs,” emphasizing that cohesive strategies that prioritize transparency, accountability and trust including information management can enhance team processes and scientific progress [17]. Furthermore, Lotrecchiano et al. defined core competencies for team science, comprising 5 personal and 8 team-related skills [7].

However, the literature on team science specifically for CRPs, such as the CRAs and CRCs in our study, has been limited. Mendell *et al.* recently addressed this gap by adapting Lotrecchiano’s framework to identify 59 “smart skills” and related skills tailored to CRPs [6]. Mendell argues that developing team science skills for CRPs may contribute to more effective collaborations across interdisciplinary clinical research teams [6]. Our study provides practical evidence supporting this perspective. The insights extracted from participant responses – such as comprehension of workflows and role division, and appreciation for professional priorities – directly align with many of Mendell’s CRP smart skills. These include “Roles and responsibilities,” “Learning & communication styles,” “Organizational structure,” “Communication methods,” “Support vision,” “Intellectual humility,” “Diverse perspectives,” “Knowledge sharing & problem solving,” “Mutual respect,” and “Open communication.” Notably, our qualitative analysis revealed that participants gained insights related to “Intellectual humility” (e.g., acknowledging others’ perspectives with empathy) and “Mutual respect,” even though these concepts were not explicit components of the training curriculum. This suggests that the experiential, on-site nature of the program organically fostered these crucial competencies. Thus, viewed from a team science perspective, this reciprocal on-site training indicates considerable potential as a practical intervention to strengthen sponsor-site collaboration.

## Limitations

This study has several limitations. First, the training program was implemented between a single large pharmaceutical company and a university hospital where clinical trials are primarily managed by in-house CRCs. Moreover, the training was conducted under a confidentiality agreement, which may limit its replicability in institutions unable to establish such agreements.

Second, the study relied on self-reported survey data, which may be subject to social desirability and recall bias. Participants’ assessments of their understanding and impressions were inherently subjective and may not accurately reflect actual knowledge or long-term attitudinal change.

Third, this study has the potential for self-selection bias. Because participation in the training was voluntary, these participants may have entered the study with greater frustrations and/or stronger motivation for improvement regarding sponsor-site interactions than those who did not volunteer.

Fourth, because the surveys were anonymous and open-ended responses could not be linked to specific job titles, we were unable to perform sub-analyses strictly limited to participants currently holding the title of CRA or CRC. Additionally, because the follow-up survey was independent of the earlier surveys, we could not assess whether baseline characteristics differed between those who completed the follow-up and those who did not, potentially introducing self-selection bias in the behavioral change results. However, given that all sponsor participants had CRA experience and nearly all site participants had CRC experience, we believe the aggregated results accurately reflect the perspectives of these professional roles.

Fifth, the effectiveness of the training was not evaluated using objective quantitative indicators such as time to query resolution, frequency of protocol deviations, trial performance metrics, or staff turnover rate. Future studies should consider incorporating such indicators.

Lastly, given the exploratory nature of this study, we did not apply a formal alpha correction for multiple comparisons in the survey analysis. While we treated each item as an independent evaluation, this approach theoretically increases the risk of Type I errors.
